# Vitamin D and its association with symptom severity in knee osteoarthritis: a cross sectional study at a national referral hospital in Uganda

**DOI:** 10.1186/s41927-021-00228-w

**Published:** 2021-12-27

**Authors:** Fiona Namutebi, James Kayima, Mark Kaddumukasa

**Affiliations:** 1 Department of Medicine, School of Medicine, College of Health Sciences, Makerere University, P. O Box 7062, Kampala, Uganda; 2 Uganda Heart Institute, Kampala, Uganda

**Keywords:** Vitamin D levels, Osteoarthritis, Uganda

## Abstract

**Background:**

Vitamin D deficiency is highly prevalent among patients with osteoarthritis. It is associated with joint pain, stiffness and worse physical function. Whether vitamin D deficiency is associated with osteoarthritis is controversial. We investigated serum vitamin D levels and its association with symptom severity in patients with knee osteoarthritis.

**Methods:**

Between January 2020 to March and May 2020, we conducted a cross sectional study at a national referral hospital in Uganda. Using the American College of Rheumatology clinical criteria, 107 consenting adults were diagnosed with knee osteoarthritis. A questionnaire captured patient demographics and clinical characteristics. Joint pain, stiffness and physical function severity were assessed and graded based on the Western Ontario and McMaster Universities Arthritis Index (WOMAC). We determined serum vitamin D levels by electrochemilumniscence immunoassay. The data were analysed and adjusted for age, sex, education, occupation, family history, body mass index (BMI) and calcium supplementation.

**Results:**

Of the 107 patients, 92 (86%) patients were females, mean (SD) age was 58.1 (12.6) years. Nearly 65% of the patients had suboptimal serum vitamin D levels < 30 ng/ml. The median (Q1, Q3) WOMAC joint scores were as follows: pain 8.0 (5, 11), stiffness 1 (0, 2), physical function 29.0 (16, 41) and total WOMAC 39.0 (21, 54). Spearman correlations between serum vitamin D levels with symptom severity were as follows: joint pain (*r* = 0.18, *p* = 0.06), stiffness (*r* = 0.13, *p* = 0.17), physical function (*r* = 0.09, *p* = 0.36) and total WOMAC (*r* = 0.13, *p* = 0.19).

**Conclusion:**

Serum vitamin D levels are not associated with joint pain, stiffness and physical function severity. Older age and higher BMI are associated with vitamin D deficiency in patients with knee osteoarthritis attending a national referral hospital rheumatology clinic in Uganda. Suboptimal vitamin D is an independent risk factor for total mortality in the general population. Clinical guidelines and further studies to determine age and BMI ranges required for vitamin D screening are needed in patients with osteoarthritis in Uganda. Patients are advised to keep a normal BMI.

## Background

Vitamin D deficiency is worryingly highly prevalent among patients with osteoarthritis and is associated with worsening joint pain [1, 2] and functional decline [3]. Vitamin D reduces bone turnover and cartilage degradation, thus potentially preventing the development and progression of osteoarthritis [4]. Osteoarthritis formerly known as a degenerative ‘wear and tear’ joint process involving cartilage and the underlying bone, is now considered as an inflammatory joint disease [5]. It is a common musculoskeletal disease worldwide and leads to functional decline, loss in quality of life and disability [6, 7]. Globally 9.6% of men and 18.0% of women aged over 60 years have symptomatic osteoarthritis. Eighty percent of those with osteoarthritis have limitations in movement and 25% cannot perform their major daily activities of life [8].

It is thought that vitamin D supplementation might be a cost effective strategy for controlling symptoms and inducing structural improvement in patients with knee osteoarthritis but the evidence is conflicting [9]. Vitamin D treatment in patients with knee osteoarthritis improved both knee pain and function [10] and may have a role in reducing knee and hip pain in the elderly with lower serum vitamin D concentrations (< 25 nmol/L) than at higher levels. On the other hand, there was no benefit of vitamin D supplementation in improving pain, stiffness, physical function and cartilage volume loss reduction [9, 11–14].

Evidence regarding serum vitamin D levels and the relationship to symptom severity in patients with osteoarthritis is lacking in Uganda yet they may potentially guide vitamin D supplementation. We determined the serum vitamin D levels and their relationship with symptom severity in patients with osteoarthritis at a National Referral Hospital.

## Methods

### Study design and setting

This was a cross sectional study that was conducted from January 2020 to March and May 2020. The study took place in the Rheumatology outpatient clinic of a 200-bed capacity national referral hospital in Kampala, Uganda. Uganda is an equatorial country that receives 12 h of sunshine daily. Majority of the people wear light clothing and spend 8–10 h outdoors [15]. The Rheumatology clinic assesses patients with joint, muscle and autoimmune diseases, and runs once a week attending to approximately 15–20 patients on each clinic day. Three quarters of these are patients with osteoarthritis. Determination of serum vitamin D levels was done at Mulago National Referral Hospital clinical chemistry laboratory. Permission was sought from the study site to transfer the blood samples to for analysis.

### Participants’ enrolment and study definitions

**Fig. 1:**
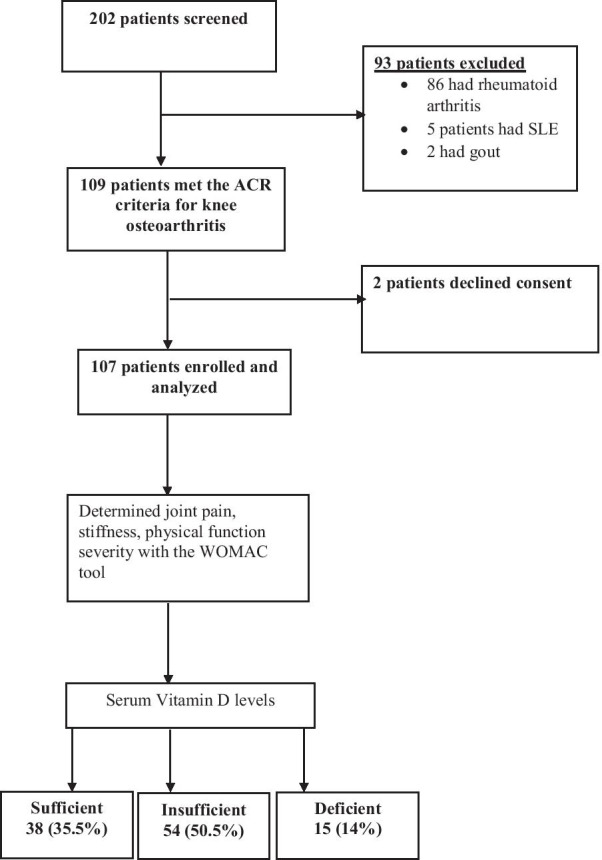
Study flow diagram

For screening, files were reviewed and patients with a diagnosis of osteoarthritis were approached for informed consent. New patients with knee pain for at least 6 months were also approached for informed consent. Adult patients (aged ≥ 18 years) with knee osteoarthritis diagnosed using the American College of Rheumatology (ACR) clinical criteria for knee osteoarthritis [16] were consecutively enrolled into the study (Fig. 1). The ACR clinical criteria for knee osteoarthritis [16] includes patients with knee pain for at least 6 months and at least three of the following six items: age > 50 years, morning stiffness of short duration < 30 min, crepitus on knee range of motion (ROM), bony tenderness, no palpable warmth and bony enlargement.

We excluded patients with a known diagnosis of rheumatoid arthritis, systemic lupus erythematosus and gout.

Patients’ serum vitamin D levels were categorized as sufficient (≥ 30 ng/ml), insufficient (20–29 ng/ml), deficient (< 20 ng/ml) based on the Endocrine Society Clinical Practice Guidelines on evaluation, treatment and prevention of vitamin D deficiency [17]. The categories insufficient and deficient were operationally defined and categorised as suboptimal.

### Data collection

After obtaining informed consent, we administered a pretested questionnaire to obtain socio-demographic characteristics, history of chronic illnesses, drugs including vitamin D and calcium supplementation, duration of daily sunshine exposure, prior or current involvement in sports, alcohol and smoking. Weight and height were taken using a calibrated weighing scale and stadiometer respectively for body mass index calculation.

#### Assessment of osteoarthritis symptom Severity (joint pain, stiffness and physical function)

Joint pain, stiffness and physical function were assessed by the Western Ontario and McMaster Universities Osteoarthritis Index (WOMAC) [18]. The WOMAC tool consists of 24 items divided into 3 subscales each with a score ranging from 0 to 4 where 0 = none, 1 = slight, 2 = moderate, 3 = severe, 4 = extreme [18]. The total WOMAC score ranges from 0 to 96 and was determined by the sum of the pain, stiffness and physical function scores. Higher scores indicate more severe disease.

The 3 subscales assessed:

1. Pain (5 items): during walking, climbing stairs, sleeping at night (in bed), resting (sitting or lying), and standing upright.
2. Stiffness (2 items): after first waking (morning) and later in the day (evening).
3. Physical Function (17 items): descending stairs, ascending stairs, rising from sitting, standing, bending to floor, walking on even floor, getting in/out of a car, shopping, putting on socks, taking off socks, rising from bed, lying in bed, getting in / out of bath, sitting, getting on/off toilet, doing light domestic duties, doing heavy domestic duties.

### Laboratory analysis

#### Sample collection

We drew about 4 mls of venous blood from each consented study patient aseptically, into a plain blood sample tube (BD Vacutainer) and allowed the blood to coagulate at room temperature for not more than 4 h. The blood samples were then taken to a clinical chemistry laboratory at the study site, centrifuged immediately and stored at − 20 °C in a refrigerator. Upon collection of an ample number of blood samples, the stored serum was transported from the study site in a cooler box with ice packs to Mulago National Referral Hospital clinical chemistry laboratory within one and half hours for further storage at − 20 °C in a refrigerator until testing for up to 3 months.

#### Diagnostic testing

Serum Vitamin D concentrations were measured on thawed sera by electrochemilumniscence immunoassay using a Cobas-6000 machine supplied by Roche Diagnostics. It measures the serum vitamin D (25 (OH) D) concentrations in the range of 4–100 ng/ml.

Measurement of albumin, calcium concentration was also done. We corrected serum calcium levels for albumin for deranged albumin levels using the following formula: Corrected calcium (mmol/L) = measured serum calcium + [(normal albumin − patient’s albumin) × 0.02]. Hypocalcaemia was defined as serum calcium concentration corrected for albumin < 2.2 mmol/L.

### Statistical analysis

Data was entered into entered into SPSS version 21 and excel and exported to STATA 15 software for analysis. Participant characteristics were summarized into means and standard deviations for continuous variables that were normally distributed and medians with their 25th and 75th percentiles for continuous variables that were not normally distributed. Patient demographic and clinical characteristic were summarized as frequencies and proportions. Using a mean pain, stiffness and physical function severity score (total WOMAC score) of 57.2 determined by the WOMAC tool with a standard deviation of 17.6 from a cross-sectional study carried out in patients with osteoarthritis in Turkey [9], sample size estimation for mean in one group was used for sample size calculation. A sample size of 107 patients was obtained and it powered the study to 80%.

The distribution of serum vitamin D levels was summarized as mean and its standard deviation because it was normally distributed. Joint pain, stiffness, physical function and total WOMAC scores were summarized into medians with their 25th and 75th percentiles because they were not normally distributed. The Spearman’s rank correlation was used to see the relationship between joint pain, stiffness, physical function, total WOMAC score and serum vitamin D levels. The coefficients were considered statistically significant, if they had a *p* value < 0.05.

### Study approval

Ethical clearance to carry out the study was obtained from the Department of Medicine Makerere University College of Health Sciences and the School of Medicine Research and Ethics Committee; Ref No: 2020-015. All study participants provided written informed consent before enrolment into the study. The result was explained to the patient/caretaker by telephone and suboptimal serum vitamin D levels were replaced.

## Results

### Baseline and clinical characteristics of the study population

We studied patients aged 18 years and above. Mean (SD) age was 58.1 (12.6). None of the patients was on vitamin D supplementation. A total of 22 (20%) patients didn’t take vitamin D containing foods i.e. milk, eggs and fish. The mean (SD) of serum vitamin D in this population was 28.3 (9.1) ng/ml. A total of 69 (64.5%) patients had suboptimal serum vitamin D levels < 30 ng/ml (Table 1).

**Table 1:** Socio-demographic, clinical and laboratory characteristics of the 107 patients

Variable	Frequency (N = 107) n	Percentage (%)
*Age in years*		
> 50	83	77.6
< 50	24	22.4
*Sex*		
Female	92	86
Maile	15	14
*Occupation*		
Employed	56	52.3
Clinical characteristics		
*Family history of osteoarthritis*		
Yes	41	38.3
*Chronic disease*		
Diabetes	60	56.1
Hypertension	16	15
*Calcium supplementation*		
No	98	91.6
*Vitamin D supplementation*		
No	107	100
*Body mass index*		
Overweight (25–29.9)	34	31.8
Obese (≥ 30)	52	48.6
Laboratory characteristics		
*Serum calcium levels*		
Sufficient	88	82.2
*Serum vitamin D (ng/ml)*		
Sufficient (≥ 30)	38	35.5
Insufficient (20–29)	54	50.5
Deficient (< 20)	15	14.0

### Disease severity

The median joint pain, stiffness, physical function and total WOMAC scores were below 50% of the maximum subscale scores (Table 2).

**Table 2:** Medians (Q1, Q3) of the different patients’ scores for joint pain, stiffness and physical function

WOMAC score	Median (Q1, Q3)	Min, max
Pain	8.0 (5, 11)	0, 19
Stiffness	1 (0, 2)	0, 8
Physical function	29.0 (16, 41)	3, 68
Total joint, stiffness, physical function (total WOMAC)	39.0 (21, 54)	4, 95

*WOMAC* Western Ontario and McMaster Universities

Joint specific pain scores range from 0 to 20 with 0 indicating no pain; joint stiffness scores range from 0 to 8 with 0 indicating no stiffness and physical function scores range from 0 to 68, with 0 indicating no difficulty with activity. The total WOMAC score is out of 96

### Association between serum vitamin D and symptom severity

There was no association between serum vitamin D and joint pain, stiffness and physical function severity. The Spearman’s rank correlation coefficients were weak and not statistically significant (Table 3).

**Table 3:** Spearman’s rank correlation coefficient of the association between serum vitamin D and joint symptoms

WOMAC score	r_s_	95% CI	*p* Value
Pain	0.18	− 0.01, 0.36	0.0575
Stiffness	0.13	− 0.06, 0.31	0.1745
Physical function	0.09	− 0.10, 0.28	0.3609
Total joint, stiffness, physical function (total WOMAC)	0.13	− 0.06, 0.31	0.1893

*WOMAC* Western Ontario and McMaster Universities

### Bivariate and Multivariate factors associated with disease severity

At bivariate analysis vitamin D was not associated with disease severity but participants’ age, albumin levels and education status were the covariates associated with disease severity. At multivariate level after adjusting for age, albumin levels and education status, sufficient serum vitamin D was found to be associated with higher disease severity when compared to deficient levels. For every one year increase in age, disease severity increases by 0.37. Higher education status compared with no education and male sex compared to female sex were associated with a lower disease severity. A unit increase in albumin is associated with a decrease in disease severity by 1.04. The total WOMAC score represents disease severity (Table 4).

**Table 4:** Bivariate and multivariate analysis of factors associated with the total WOMAC score

Variable	Crude B	*p* Value	Adjusted B	95% CI	*p* Value
*Vitamin D*					
Deficient	1				
Sufficient	6.44	0.307	12.75	1.13, 24.37	0.032
Insufficient	6.01	0.401	5.98	− 4.94, 16.89	0.28
*Age in years*	0.43	0.005	0.37	0.06, 0.69	0.019
*Sex*					
Female	1				
Male	− 9.2	0.107	− 11.55	− 22.05, − 1.06	0.031
*Education*					
None	1				
Primary	− 20.67	0.004	− 16.18	− 30.36, − 2.01	0.026
Secondary	− 26.53	< 0.001	− 18.33	− 33.32, − 3.33	0.017
Tertiary	− 24.4	0.004	− 13.46	− 30.37, 3.45	0.117
*Occupation*					
Employed	1				
Unemployed	2.87	0.472			
*Chronic diseases*					
No	1				
Yes	− 3.2	0.466			
*Calcium supplementation*					
No	1				
Yes	9.68	0.176			
*Family history*					
No	1				
Yes	2.65	0.518			
*Sport*					
No					
Yes	− 3.08	0.44			
*BMI*	0.19	0.551			

### Association between age and Serum vitamin D

Younger patients with mean age (SD) 54.0 (13.7), *p* value 0.04, were found to have sufficient serum vitamin D levels compared to older patients. A lower BMI (SD) of 28.3 (6.7), *p* value (0.039) was associated with sufficient serum vitamin D levels compared to higher BMIs (Table 5).

**Table 5:** Association between age, BMI and serum vitamin D

Variable	Deficient	Sufficient	Insufficient	*p* Value
Age in years, mean (SD)	61.4 (10.2)	54.0 (13.7)	60.0 (11.9)	0.040
BMI, mean (SD)	32.2 (6.4)	28.3 (6.7)	31.2 (5.7)	0.039

*p Value is from one-way ANOVA

## Discussion

The main findings of our study are; (1) nearly 65% of the study participants had suboptimal serum vitamin D levels (< 30 ng/ml) and (2) no significant correlation between serum vitamin D levels and the severity of joint pain, stiffness and physical function. Older age was found to be associated with higher disease severity. Higher education status, male sex and higher albumin levels were associated with a lower disease severity. Younger age and a lower BMI were associated with sufficient serum vitamin D levels.

Overall, the mean serum vitamin D levels in our study patients were insufficient at 28.3 (9.1) ng/ml. This may be due to low dietary intake of vitamin D, obesity or black race. However, the serum vitamin D level in our study was not as low as in other studies probably because Uganda lies along the equator and receives sunshine all year round. Mean (SD) baseline serum vitamin D concentrations in patients with osteoarthritis in different geographical locations ranged from 22.4 (9) ng/ml in Malawi Sub-Saharan Africa [19] to 11.57 (8.98) ng/ml in Turkey [9]; 15 (3.01) ng/ml in India [10] and 20.7 (8.9) ng/ml in UK [11]. However, we didn’t explore the role/type of cloth coverings that the study participants use as these might reduce the surface area for sun exposure.

All joint symptoms in our study were below 50% of the maximum subscale scores indicating less disabling disease. This could be either because the disease was well controlled, or that genetics, seasonal variations and cultural differences may have a role to play in the way patients perceive joint symptoms.

There was no correlation between serum vitamin D levels and the severity of joint symptoms suggesting that other mechanisms could be involved in the pathogenesis of osteoarthritis or simply that osteoarthritis is a degenerative disease not related to vitamin D in our settings. This finding is similar to a study in Turkey where 90% of the patients with knee osteoarthritis were vitamin D deficient (< 10 ng/ml) [9]. On the other hand, relatively low serum vitamin D concentrations were associated with higher joint pain scores in a cross sectional study among postmenopausal women in the USA [20]. Low vitamin D levels were also associated with pain in a cross sectional study of a large-scale population from the Hertfordshire cohort study in United Kingdom [2]. Moderate vitamin D deficiency independently predicted incident, or worsening knee and hip pain over 5 years in a longitudinal population-based cohort study of randomly selected older adults in Australia [1]. There is paucity of data comparing serum vitamin D concentrations and physical function severity in patients with knee osteoarthritis using the WOMAC index. Our study found no correlation between serum vitamin D concentrations and physical function. This is in keeping with a study in Turkey where 84.1% of the study population was vitamin D deficient [21] and in Kuwaiti patients where vitamin D deficiency was prevalent at 92.9% [22]. These two studies however used other tools to assess physical function. Our study results showed no correlation between serum vitamin D concentrations and joint stiffness. There is also paucity of data comparing serum vitamin D concentrations and stiffness probably because joint stiffness in patients with osteoarthritis is usually of a shorter duration i.e. < 30 min as compared to ≥ 1 h in patients with inflammatory arthritis and therefore might not be a major complaint.

Addressing factors that may impact vitamin D synthesis such as reduced outdoor activities, and type of clothing which reduces the required ultraviolet-B (UVB)-induced vitamin D production in the skin especially in dark skinned people is required. People with a naturally dark skin tone have natural sun protection and require at least three to five times longer exposure to make the same amount of vitamin D as a person with a white skin tone [23]. Increased skin pigment reduces the capacity of skin to synthesize vitamin D3. A higher prevalence of vitamin D deficiency is associated with immigrant background among children and adolescents in Germany [24]. Also we need to think about possible medications which interfere with vitamin D production in our population like anticonvulsants and HAART. Steroid and xenobiotic receptor and vitamin D receptor crosstalk mediates CYP24 expression and drug-induced osteomalacia [25]. This high prevalence of suboptimal vitamin D poses a particularly important public health issue as it’s an independent risk factor for total mortality in the general population [26].

This study is not without its limitations. The relatively small sample size in addition to being a hospital-based setting and its cross-sectional design may not allow for generalization of these findings to the population of Ugandan participants with osteoarthritis. A community-based designed study with a larger sample size might be able to address these concerns. However, the strength of our study lies in the fact that we have recruited a quite heterogeneous group of patients with osteoarthritis. The dietary intake and medication details were not actively explored. Also due to the cross-sectional design of the study, a temporal relationship between suboptimal vitamin D levels and osteoarthritis could not be established.

## Conclusion

Serum vitamin D levels are not associated with joint pain, stiffness and physical function severity. Older age and a higher BMI are associated with vitamin D deficiency in patients with knee osteoarthritis attending a national referral hospital rheumatology clinic in Uganda. Suboptimal vitamin D is an independent risk factor for total mortality in the general population. Clinical guidelines and further studies to determine age and BMI ranges required for vitamin D screening are needed in patients with osteoarthritis in Uganda. Patients are advised to keep a normal BMI.

## Data Availability

Data and materials used in this study are available from the corresponding author on reasonable request.

## Abbreviations

ACR: American College of Rheumatology
BMI: Body mass index
WOMAC: Western Ontario and McMaster Universities Osteoarthritis Index

## References

[1] Laslett LL, Quinn S, Burgess JR, Parameswaran V, Winzenberg TM, Jones G, et al. Moderate vitamin D deficiency is associated with changes in knee and hip pain in older adults: a 5-year longitudinal study. Ann. Rheumatic Dis. 2013:annrheumdis-2012-202831. 10.1136/annrheumdis-2012-202831 23595144

[2] Muraki S, Dennison E, Jameson K, Boucher B, Akune T, Yoshimura N (2011). Association of vitamin D status with knee pain and radiographic knee osteoarthritis. Osteoarthr Cartil.

[3] Sohl E, Van Schoor N, De Jongh R, Visser M, Deeg D, Lips P (2013). Vitamin D status is associated with functional limitations and functional decline in older individuals. J Clin Endocrinol Metab.

[4] McAlindon TE, Felson DT, Zhang Y, Hannan MT, Aliabadi P, Weissman B (1996). Relation of dietary intake and serum levels of vitamin D to progression of osteoarthritis of the knee among participants in the Framingham Study. Ann Intern Med.

[5] Liu-Bryan R, Terkeltaub R (2015). Emerging regulators of the inflammatory process in osteoarthritis. Nat Rev Rheumatol.

[6] Guccione AA, Felson DT, Anderson JJ, Anthony JM, Zhang Y, Wilson P (1994). The effects of specific medical conditions on the functional limitations of elders in the Framingham Study. Am J Public Health.

[7] Neogi T (2013). The epidemiology and impact of pain in osteoarthritis. Osteoarthr Cartil.

[8] Chronic Diseases and Health Promotion [Internet]. http://www.who.int/chp/topics/rheumatic/en/.

[9] Cakar M, Ayanoglu S, Cabuk H, Seyran M, Dedeoglu SS, Gurbuz H (2018). Association between vitamin D concentrations and knee pain in patients with osteoarthritis. PeerJ.

[10] Sanghi D, Mishra A, Sharma AC, Singh A, Natu S, Agarwal S (2013). Does vitamin D improve osteoarthritis of the knee: a randomized controlled pilot trial. Clin Orthop Relat Res.

[11] Arden NK, Cro S, Sheard S, Doré CJ, Bara A, Tebbs SA (2016). The effect of vitamin D supplementation on knee osteoarthritis, the VIDEO study: a randomised controlled trial. Osteoarthr Cartil.

[12] Jin X, Jones G, Cicuttini F, Wluka A, Zhu Z, Han W (2016). Effect of vitamin D supplementation on tibial cartilage volume and knee pain among patients with symptomatic knee osteoarthritis: a randomized clinical trial. JAMA.

[13] McAlindon T, LaValley M, Schneider E, Nuite M, Lee JY, Price LL (2013). Effect of vitamin D supplementation on progression of knee pain and cartilage volume loss in patients with symptomatic osteoarthritis: a randomized controlled trial. JAMA.

[14] Alsubiaee KM (2016). The effects of vitamin D supplementation in patients with knee osteoarthritis: uncontrolled open label clinical trial. J Arthritis.

[15] Nansera D, Graziano F, Friedman D, Bobbs M, Jones A, Hansen K (2011). Vitamin D and calcium levels in Ugandan adults with human immunodeficiency virus and tuberculosis. Int J Tuberc Lung Dis.

[16] Altman R, Asch E, Bloch D, Bole G, Borenstein D, Brandt K (1986). Development of criteria for the classification and reporting of osteoarthritis: classification of osteoarthritis of the knee. Arthritis Rheum Off J Am Coll Rheumatol.

[17] Holick MF, Binkley NC, Bischoff-Ferrari HA, Gordon CM, Hanley DA, Heaney RP (2011). Evaluation, treatment, and prevention of vitamin D deficiency: an Endocrine Society clinical practice guideline. J Clin Endocrinol Metab.

[18] Bolognese JA, Schnitzer T, Ehrich E (2003). Response relationship of VAS and Likert scales in osteoarthritis efficacy measurement. Osteoarthr Cartil.

[19] Allain TJ, Beresford PA, Newman JH, Swinkels A (2008). Vitamin D levels in patients undergoing knee arthroplasty: does vitamin D status effect postoperative outcomes?. e-SPEN Eur e-J Clin Nutr Metabol.

[20] Chlebowski RT, Johnson KC, Lane D, Pettinger M, Kooperberg CL, Wactawski-Wende J (2011). 25-Hydroxyvitamin D concentration, vitamin D intake and joint symptoms in postmenopausal women. Maturitas.

[21] Başkan BM, Yurdakul FG, Aydın E, Sivas F, Bodur H (2018). Effect of vitamin D levels on radiographic knee osteoarthritis and functional status. Turk J Phys Med Rehabil..

[22] Al-Jarallah KF, Shehab D, Al-Awadhi A, Nahar I, Haider MZ, Moussa MA (2012). Are 25 (OH) D levels related to the severity of knee osteoarthritis and function?. Med Princ Pract.

[23] Clemens T, Henderson S, Adams J, Holick M (1982). Increased skin pigment reduces the capacity of skin to synthesise vitamin D3. The Lancet.

[24] Hintzpeter B, Scheidt-Nave C, Müller MJ, Schenk L, Mensink GB (2008). Higher prevalence of vitamin D deficiency is associated with immigrant background among children and adolescents in Germany. J Nutr.

[25] Zhou C, Assem M, Tay JC, Watkins PB, Blumberg B, Schuetz EG (2006). Steroid and xenobiotic receptor and vitamin D receptor crosstalk mediates CYP24 expression and drug-induced osteomalacia. J Clin Investig.

[26] Melamed ML, Michos ED, Post W, Astor B (2008). 25-hydroxyvitamin D levels and the risk of mortality in the general population. Arch Intern Med.

